# Perioperative Anaesthetic Approaches to Paediatric Patients: A National Survey

**DOI:** 10.4274/TJAR.2025.252053

**Published:** 2025-05-30

**Authors:** Ayşe Çiğdem Tütüncü, Zehra Hatipoğlu, Halil Cebeci, Elif Çopuroğlu, Dilek Altun, Serpil Zehra Ustalar Özgen

**Affiliations:** 1 İstanbul University-Cerrahpaşa, Cerrahpaşa Faculty of Medicine, Department of Anaesthesiology and Intensive Care, İstanbul, Türkiye; 2 Çukurova University Faculty of Medicine, Department of Anaesthesiology and Reanimation, Adana, Türkiye; 3 Ondokuz Mayıs University Faculty of Medicine, Department of Anaesthesiology and Reanimation, Samsun, Türkiye; 4 Trakya University Faculty of Medicine, Department of Anaesthesiology and Reanimation, Edirne, Türkiye; 5 Acıbadem Mehmet Ali Aydınlar University Vocational School of Health Services, Bakırköy Acıbadem Hospital, Clinic of Anaesthesiology and Reanimation, İstanbul, Türkiye; 6 Acıbadem Mehmet Ali Aydınlar University Faculty of Medicine, Department of Anaesthesiology and Reanimation, İstanbul, Türkiye

**Keywords:** Anaesthesia, national survey, paediatric, perioperative, postoperative, preoperative

## Abstract

**Objective:**

This study aims to assess the practices of anaesthesiologists in Türkiye regarding paediatric anaesthesia. It focuses on preoperative, intraoperative, and postoperative care protocols.

**Methods:**

Survey data were collected using a web-based electronic platform. The participants were asked to answer the questions based on the available equipment in their hospitals in daily practice. The questionnaire forms were sent to participants by the Turkish Society of Anesthesiology and Reanimation via e-mail

**Results:**

Three hundred five anaesthesiologists responded to the survey. The specific practices and standards for paediatric anaesthesia in Türkiye along with how anaesthesiologists approach paediatric patients were concluded from the survey results.

**Conclusion:**

There are still gaps in paediatric anaesthesia practice. We believe that further research and dedicated discussions on this topic will play a key role in addressing these drawbacks.

**Main Points**

• This study highlights the choices of anaesthesiologists in Türkiye regarding the preoperative, perioperative and postoperative periods in paediatric patients.

## Introduction

Newborns, infants, and children differ from adults in their anatomy, physiology, pharmacology, emotions and social interactions. Moreover, they differ from each other in terms of these features. These fundamental characteristics make paediatric anaesthesia for individuals under the age of 18 unique.

Even healthy children may require surgical or diagnostic interventions for their medical care at some point in their lives, the anaesthesia procedures required for these children hold an important place in daily practice. It is known that, on average, one out of every seven children receives general anaesthesia at least once before the age of 3.^1^

The development of new monitoring devices, updates in anaesthesia protocols, increased use of minimally invasive surgeries, and advances in postoperative care are guiding us as anaesthesiologists in our professional development. In this context, the characteristics of paediatric anaesthesia in Türkiye, the approach of anaesthesiologists to these patients, and the infrastructure of hospitals are not fully defined. This study aims to evaluate the approach of anaesthesiologists to paediatric anaesthesia in Türkiye, focusing on preoperative, intraoperative, and postoperative care.

## Methods

The study was approved by the Çukurova University Faculty of Medicine Non-Interventional Clinical Research Ethics Committee (decision no.: 12/129, and date: January 6, 2023). The cross-sectional survey-based study was conducted between February and March 2023. Survey data were collected using a web-based electronic platform. First, the research team of seven anaesthesiologists discussed the content and created the structure. Then, a pilot study was conducted on twenty anaesthesia trainees. The research team reviewed the necessary corrections of the questionnaire, and the questionnaire was finalized. The participants were asked to answer the questions based on the equipment available in their hospitals in daily practice.

The questionnaire forms were sent to participants by the Turkish Society of Anesthesiology and Reanimation via e-mail. Simultaneously, the authors informed participants in nearby provinces again via WhatsApp.

Since the relevant article is a survey study, patient consent is not required.

### Statistical Analysis

Statistical analysis was performed using the SPSS version 20.0 statistical software package (IBM SPSS Statistics for Windows, Version 20.0; IBM Corp., Armonk, New York, USA). Descriptive analysis, including frequency (%), mean ± standard deviation (and, if necessary, median, minimum and maximum), was used for statistical analysis.

## Results

Three hundred and five anaesthesiologists responded to the survey. Demographic characteristics of participants have been presented in Table 1.

When the general characteristics are examined, 76% of the respondents indicated that the youngest age group was neonates. Additionally, 46.4% indicated that they administered anaesthesia to approximately 50 paediatric patients per month, and they administered anaesthesia to the 1-6 age group most frequently in their daily practice, with an average frequency of 30%. While American Society of Anesthesiologists (ASA) I-II group paediatric patients constituted more than 50% of the daily practice, ASA III-IV group patients accounted for less than 10% (Table 2). The majority of participants reported that daily anaesthesia practice consists of minor surgeries. 51.1% of surgical cases were from the Ear, Nose, and Throat (ENT) department (Table 3).

### Preoperative Period

59.3% of the respondents reported no preoperative unit in their operating rooms. According to the survey results, the premedication usage rate in paediatric patients was 53.8%. The rate of acceptance for parents or guardians in the preoperative unit was 57.8% (Table 4).

### Intraoperative Period

During minor surgeries, standard ASA monitoring methods, excluding temperature, were commonly employed. While invasive arterial blood pressure monitoring was most frequently used in addition to routine ASA monitoring in major surgeries, cardiac output (CO) monitoring was used the least frequently (Table 4).

The rate of intravenous agent use during anaesthesia induction was 65.6%, and for anaesthesia maintenance, sevoflurane, neuromuscular blockers, fentanyl, and remifentanil were the most commonly chosen options. The most commonly preferred fluids in the perioperative period were 0.9% NaCl as the crystalloid solution and hydroxyethyl starch (HES) 130/0.4 as the colloid solution. The internal jugular vein was the most frequently catheterized vein. During catheterization, the ultrasound (US) usage rate was similar to that of the landmark technique.

### Respiratory Management

The preferred modes of mechanical ventilation were as follows: pressure control ventilation (69.3%), volume control ventilation (68.9%), pressure support ventilation (16.9%), and spontaneous ventilation (7.9%). The most commonly applied values of mechanical ventilator parameters were as follows: 6-8 mL kg^-1^ for tidal volume (Vt), 5 cmH_2_O for positive end-expiratory pressure (PEEP), and 40-49% for inspired oxygen concentration. The recruitment maneuver usage rate among respondents was 58.7%. The usage rate of the open system for anaesthesia was 17.8% (Table 5).

### Postoperative Period

Sixty-one percent of participants stated that they kept their patients waiting for less than 30 minutes in the postoperative unit. Other answers related to the postoperative period are presented in Table 5.

Seventy point four percent of the respondents were using the regional analgesia technique less frequently. Among the various regional techniques used, the epidural method was the most common (63.2%). In the early postoperative period, the most commonly used intravenous analgesic options were fentanyl (83.8%) and morphine (83.1%). The CHEOPS and FLACC scores ranked first and second, respectively, in pain assessment (Table 6).

## Discussion

In this study, we described the perioperative anaesthesia approaches used by anaesthesiologists in Türkiye for children of all age groups. To the best of our knowledge, this study is the first nationwide survey on paediatric anaesthesia. The results of the study show that paediatric patients in anaesthesia practice represent a minor proportion (<30%) of daily practice in our country, and most of these patients were >1 year old and underwent minor surgery.

In the article published in 2008, it was stated that according to data obtained from the World Health Organization, 30% of all anaesthesia procedures in daily practice occur in children under the age of 15.^2^ This study revealed that paediatric anaesthesia accounts for a substantial portion, ranging from 10% to 30%, of daily anaesthesia practices in our country. The majority of this rate includes children aged 1 to 6 years with ASA I-II classification. Similarly, in the study by Bartels et al.,^3^ where they examined 2,473,411 anaesthetics in patients aged <18 years, the majority of cases were children with ASA I-II classification. Consistent with the literature, our results showed that the ENT department had the highest frequency of surgeries.^4^ The next most common surgical specialty was orthopedics and traumatology. The data from the research indicate that minor surgeries were primarily performed on children.

Anxiety is a distressing phenomenon for both the child and the parent and can potentially accelerate behavioral changes in children in the postoperative period. Therefore, the establishment of dedicated preoperative units provides an appropriate environment for waiting before surgery in the operating room and facilitates the implementation of measures aimed at reducing anxiety. These interventions may include the administration of premedication, enabling parental presence during the preoperative phase, and using visual aids such as videos.^5^ However, based on the survey results, the prevalence of preoperative waiting areas is relatively low. In fact, expecting high rates of premedication or parental presence to alleviate anxiety, given the limited number of preoperative units, would be misplaced, and our results support this.

The data highlight the extensive use of standard monitoring techniques in paediatric patients. These techniques, employed concurrently with modern surgical procedures, are evolving, serving as complementary tools alongside standard monitoring practices in paediatric patients. This integration aims to enhance perioperative safety and overall patient care. When examining all monitoring techniques, such as depth of anaesthesia, neuromuscular, CO, cerebral oximetry, and invasive blood pressure (IBP) monitoring, it becomes apparent that IBP is the most frequently used. Notably, it is thought that IBP is especially prioritized because it allows close monitoring of hemodynamic balance and blood gas evaluations.

Inhalation agents have always been the first choice in anaesthesia practices for children, especially in cases where vascular access cannot be established and the child is afraid of these procedures. However, inhalation agents may cause more respiratory problems than intravenous agents. In the course of time, the use of intravenous agents during anaesthesia induction has begun to increase. Currently, both inhalation and intravenous agents can be used.^6^ According to our study, participants stated that they mostly used intravenous agents.

The most commonly used perioperative fluid choices were isotonic fluids such as 0.9% normal saline, Ringer’s lactate, and isolyte. However, the use of 5% dextrose fluids in maintenance fluids was remarkable. However, current understanding discourages the routine use of dextrose fluids due to their potential association with neuronal damage. Informative seminars may be organized on this subject. In cases where crystalloids are used but the intravascular space is still not sufficiently filled, the fluids needed to maintain intravascular volume are colloids, which can be synthetic or natural. According to the survey result, the usage of HES and gelofusine was high.^7^

A central venous catheter (CVC) in paediatrics may be required both intraoperatively and postoperatively for monitoring, shock, dehydration, difficult peripheral venous cannulation, parenteral nutrition, vasopressor therapy, and procedures involving significant blood or fluid loss.^8, 9^ There are various infusion sites including the internal jugular vein (IJV), subclavian vein, femoral vein, brachiocephalic vein, and peripheral veins. In our study, participants stated that they preferred IJV first and peripheral catheterization second. We believe that these options are preferred because of lower complication rates and a more accessible location. Two techniques can be used for CVC placement: the landmark technique and the real-time US-guided technique. Nowadays, US-guided CVC placement is widely used because it shortens the procedure time, increases the success rate, and decreases the risk of inadvertent arterial punctures.^9, 10^ Both techniques are used at the same rate in our study.

In paediatric anaesthesia practice, pressure controlled ventilation modes are more often preferred to volume controlled ventilation (VCV) modes. Previously, it has been suggested that VCV modes are avoided due to the potential for high airway pressures, and lack of confidence in the accuracy of Vt delivery.^11^ Today, it is recommended to use alternative modes to these.^12^ In essence, the primary objective is to minimize potential lung injury and ensure lung-protective ventilation with the selected mode. In this study, both ventilation modes were used, with pressure support ventilation being mentioned as the second mode. Other parameters were Vt and PEEP. The participants’ responses were generally in line with the literature. The literature recommends 6-8 mL of Vt per kg of ideal body weight for lung protective ventilation. PEEP is an essential parameter to prevent alveolar collapse, but there is no consensus on the optimal level of PEEP in children. The recommended PEEP level is within the physiological range, which is 3-5 cmH_2_O.^13, 14^ A healthy child is typically expected to have an oxygen saturation level greater than 95% when breathing room air. Similarly, there is no consensus regarding the optimal fraction of inspired oxygen (FiO_2_) value. Once the airway is secured, the minimum FiO_2_ should be maintained according to the SpO_2_ value.^13^ However, we should note that the use of low FiO_2_ values (<40%) was infrequent among participants, according to the survey findings. The survey also considers anaesthesia systems, revealing a lower ratio of efficiency associated with the use of open systems. Old anaesthesia machines for children did not have a functional ventilator, and ventilation was provided manually. However, manual ventilation may cause volutrauma, barotrauma, and atelectasis.^15^ Today, modern anaesthesia machines, equipped with ventilators, are widely used. According to the survey, manual ventilation is frequently favored in anaesthesia training and during anaesthesia machine malfunctions.

Pain management is a crucial therapeutic approach that is equally applicable to paediatric patients. The initial step in pain management involves assessing pain based on the child’s age. In our study, the most frequently utilized pain assessment tools were the Children’s Hospital of Eastern Ontario Pain Scale (for ages 1-7 years), the Face, Legs, Activity, Cry and Consolability (for ages 2 months-7 years), the Wong Baker Faces Scale (for ages 3-7 years), and the Visual Analogue Scale (for school-age children). In fact, this finding appears to be consistent with the age range typically anaesthetized in routine clinical practice.^16, 17^ Currently, a multimodal approach is being employed to mitigate the adverse effects associated with opioid agents. The multimodal approach involves the use of non-opioid medications, such as paracetamol and non-steroidal drugs, as well as non-pharmacologic treatments, such as hypnosis, massage, and heat compresses. Additionally, it includes regional techniques, such as peripheral blocks and central neuraxial blocks.^16^ However, this study observed that intravenous methods, especially opioid agents, are still preferred as the initial choice for managing certain types and severities of pain. Among regional techniques, peripheral nerve blocks are at the forefront, in parallel with the advancement of technology.

Paediatric patients are transferred to the post-anaesthesia care unit (PACU) to ensure they fully awaken, similar to adult patients. Although it is recommended to stay in the PACU at least 30 minutes, several factors can influence this duration.^18, 19^ However, the results of this study indicate that the waiting time in the PACU is generally less than 30 minutes. The lack of PACU facilities may contribute to this situation. The presence of parents with their children in the PACU may have a positive impact on the children’s postoperative behavior.^20, 21^ Evaluating the breathing, circulation, and consciousness of children planned to be discharged from PACU will play an effective role in reducing possible complications. Respondents reported using Aldrete scoring more frequently in this study.^19^

### Study Limitations

Considering the limitations of the study, since it is a survey study, the accuracy of the data will be limited to the statements of the survey participants. A larger number of survey participants would certainly increase both data diversity and accuracy.

## Conclusion

In conclusion, acknowledge that we still have shortcomings in paediatric anaesthesia practice. We believe that the studies to be conducted and the meetings to be held on this subject will help reduce these imperfections.

## Ethics

**Ethics Committee Approval:** The study was approved by the Çukurova University Faculty of Medicine Non-Interventional Clinical Research Ethics Committee (decision no.: 12/129, and date: January 6, 2023).

**Informed Consent:** Since the relevant article is a survey study, patient consent is not required.

## Figures and Tables

**Table 1. Characteristics of Participants:** 

-	**n (%)**
**Age (years)**	43.9±8.3
**Gender (Female/Male)**	208/97 (68.2/31.8)
**Position**	
Specialist doctor Assistant professor Associate professor Professor doctor	194 (63.8) 30 (9.9) 48 (15.8) 32 (10.5)
**Graduated**	
University hospital Ministry of health education and research hospital Ministry of health/university hospital	243 (79.7) 49 (16.1) 13 (4.3)
**Affiliation**	
University hospital Ministry of health education and research hospital Ministry of health/university hospital Public hospital Private hospital	82 (26.9) 61 (20.0) 39 (12.8) 67 (22.8) 56 (18.4)

**Table 2. Age Distribution of Paediatric Patients in Anaesthesia Practice:** 

-	**n (%)**
**Ratio of paediatric/adult patients in daily anaesthesia practice**	
<10% 10-30% 31-50% 51-80% 81-100%	108 (35.4) 120 (39.3) 35 (11.5) 12 (3.9) 30 (9.8)
**Paediatric age group most frequently anaesthetized in the daily practice**	
0-1 month 1 month-1 year 1-6 years 6-10 years >10 years	38 (12.5) 80 (26.2) 249 (81.6) 122 (40.0) 59 (19.3)
**The smallest patient age range you have received in your anaesthesia practice**	
<1 month 1-6 month 6 month-1 year 1-3 years >3 years	234 (76.7) 25 (8.2) 20 (6.6) 19 (6.2) 7 (2.3)
**What is the monthly count of paediatric patients receiving anaesthesia?**	
<10 11-50 51-100 >100	69 (22.7) 141 (46.4) 35 (11.5) 59 (19.4)
**Premature babies rate in daily anaesthesia practice**	
<10% 10-25% 25-50% >50%	279 (92.7) 19 (16.3) 2 (0.7) 1 (0.3)
**Number of paediatric patients with ASA I-II in daily practice**	
<10% 10-25% 25-50% >50	14 (4.6) 27 (8.9) 86 (28.2) 178 (58.4)
**Number of paediatric patients with ASA III-IV in daily practice**	
<10% 10-25% 25-50% >50	101 (33.6) 76 (25.2) 88 (29.2) 36 (12)

ASA, American Society of Anesthesiologists.

**Table 3. Surgical Procedures:** 

-		**n (%)**
**The most common surgical procedures in which anaesthesia is given in daily practice (Yes/No)**	Ear nose throat Orthopedics and traumatology Paediatric surgery Urology Outpatient anaesthesia Neurosurgery Plastic and reconstructive surgery Cardiovascular surgery Ophthalmic surgery Other	156/149 (51.1/48.9) 145/160 (47.5/52.5) 131/174 (43.0/57.0) 105/200 (34.4/65.6) 87/218 (28.5/71.5) 55/250 (18.0/82.0) 46/259 (15.1/84.9) 37/268 (12.1/87.9) 38/267 (12.5/87.5) 69/236 (22.6/77.4)
**Minor surgery rate in daily anaesthesia practice** <10% 10-25% 25-50% >50%		- 47 (15.4) 66 (21.6) 87 (28.5) 105 (34.4)
**Major surgery rate in daily anaesthesia practice** <10% 10-25% 25-50% >50%		- 84 (27.7) 66 (21.8) 88 (29.0) 65 (21.5)

**Table 4. Perioperative Applications:** 

-			**n (%)**
**Preoperative period**	Is there a paediatric preoperative unit? (Yes/No)		124/181 (40.7/59.3)
	Routine premedication (Yes/No)		164/141 (53.8/46.2)
	Acceptance of parents or guardians in the preoperative unit (Yes/No)		160/117 (57.8/42.2)
**Anaesthesia**	Monitoring methods used during minor surgery procedures (Yes/No)	ECG NIBP SpO_2_ EtCO_2_ Temperature	298/7 (97.7/2.3) 246/58 (80.9/19.1) 301/4 (98.7/1.3) 277/27 (91.1/8.9) 105/199(34.5/65.5)
	Monitoring methods used in addition to standard monitoring in major surgery procedures (Yes/No)	Depth of anaesthesia monitoring Neuromuscular monitoring Cardiac output monitoring Cerebral oximeter Invasive arterial blood pressure monitoring Other	90/187 (32.5/67.5) 40/237 (14.4/85.6) 34/242 (12.3/87.7) 77/200 (27.8/72.2) 230/47 (83.0/17.0) 80/197 (28.9/71.1)
	Induction	Intravenous agents	200 (65.6)
		Inhalation agents	65 (21.3)
		Intravenous+inhalation agents	39 (12.8)
		Inhalation+other	1 (0.3)
	Maintenance (Yes/No)	Sevoflurane	298/4 (98.7/1.3)
		Desflurane	82/220 (27.8/72.8)
		Neuromuscular blocker	220/82 (72.8/27.8)
		Fentanyl	218/83 (72.4/27.6)
		Remifentanil	228/74 (75.5/24.5)
		Dexmedetomidine	22/280 (7.3/92.7)
		Other opioids	44/258 (14.6/85.4)
**The most commonly used perioperative fluid choice**	Crystalloid solutions	0.9% NaCl	166/137 (54.8/45.2)
		Ringer lactate	77/226 (25.4/74.6)
		Isolyte	127/176 (41.9/58.1)
		5% dextrose in 0.45% NaCl	143/160 (47.2/52.8)
		5% dextrose	18/285 (5.9/94.1)
		10% dextrose	0/303 (0.0/100.0)
		Other fluids	33/271 (10.9/89.1)
	Colloid solutions	Gelofusine	87/197 (30.6/69.4)
		Hydroxyethyl starch (130/0.4)	114/170 (40.1/59.9)
		Fresh frozen plasma	69/215 (24.3/75.7)
		Albumin	14/270 (4.9/95.1)
**The most commonly used perioperative fluid choice**	Crystalloid solutions	0.9% NaCl	166/137 (54.8/45.2)
		Ringer lactate	77/226 (25.4/74.6)
		Isolyte	127/176 (41.9/58.1)
		5% dextrose in 0.45% NaCl	143/160 (47.2/52.8)
		5% dextrose	18/285 (5.9/94.1)
		10% dextrose	0/303 (0.0/100.0)
		Other fluids	33/271 (10.9/89.1)
	Colloid solutions	Gelofusine	87/197 (30.6/69.4)
		Hydroxyethyl starch (130/0.4)	114/170 (40.1/59.9)
		Fresh frozen plasma	69/215 (24.3/75.7)
		Albumin	14/270 (4.9/95.1)
**Perioperative catheterization**	Most commonly used veins	Internal jugular venous	218/77 (73.9/26.1)
		Subclavian venous	75/220 (25.4/74.6)
		Brachiocephalic venous	17/278 (5.8/94.2)
		Femoral venous	78/217 (26.4/73.6)
		Peripheral veins	145/149 (49.3/50.7)
	Most commonly used method	Landmark	147 (50.7)
		Ultrasound-guided	143 (49.3)
**Postoperative period**	Is there a paediatric postoperative unit? (Yes/No)		166/139 (54.4/45.6)
	Acceptance of parents or guardians in the postoperative unit (Yes/No)		124/165 (42.8/56.9)
	Patient’s waiting time in the postoperative unit <30 min 30-60 min >60 min		180 (61.4) 96 (32.8) 17 (5.8)
	Scoring systems in the postoperative unit Aldrete score Ped-PADSS No Other		202/96 (67.8/32.2) 64/234 (21.5/78.5) 56/240 (18.9/81.1) 15/283 (5.0/95.0)
	Use of heater (Y/N)		263/34 (88.6/11.4)

ECG, electrocardiography; NIBP, non-invasive blood pressure; SpO_2, _Peripheral oxygen saturation, EtCO_2,_ End-tidal carbon dioxide; Ped-PADSS, paediatric post-anaesthetic discharge scoring system.

**Table 5. Respiratory Management:** 

-		**n (%)**
**Volume control ventilation modes**	(Yes/No)	208/94 (68.9/31.1)
**Pressure control ventilation modes**	(Yes/No)	205/91 (69.3/30.7)
**Pressure support ventilation**	(Yes/No)	51/251 (16.9/83.1)
**Spontaneous ventilation**	(Yes/No)	24/278 (7.9/92.1)
**Other**	(Yes/No)	4/298 (1.3/98.7)
**Tidal volume**	<6 mL kg^-1^ 6-8 mL kg^-1^ 8-10 mL kg^-1^ >10 mL kg^-1^	28 (9.3) 178 (58.9) 94 (31.1) 2 (0.7)
**Use of recruitment maneuver**	(Yes/No)	175/123 (58.7/41.3)
**Use of PEEP**	(Yes/No)	241/61 (79.8/20.2)
**Table 5. Continued**		
**Value of PEEP**	1-2 3 4 5 >5	20 (8.2) 70 (28.7) 56 (23.0) 89 (36.5) 9 (3.7)
**Inspired oxygen concentration**	21-29% 30-39% 40-49% 50-99% 100%	5 (1.7) 48 (15.9) 194 (64.2) 55 (18.2) 0 (0.0)
**Use of open system**	(Yes/No)	54/249 (17.8/82.2)
**Reason for choosing manual ventilation** Causes due to anaesthesia machine Assistant training Habit Causes due to anaesthesia machine + assistant training Assistant training + habit		- 82 (60.3) 24 (17.6) 9 (6.6) 20 (14.7) 1 (0.7)

PEEP, positive end-expiratory pressure.

**Table 6. Pain Management:** 

-		**n (%)**
**Pain management**	**Regional analgesia administration rate** <25% 25-50 % 50-75 % >75%	- 209 (70.4) 52 (17.5) 20 (6.7) 16 (5.4)
	**The most preferred method of analgesia** Intravenous methods Regional methods Multimodal analgesia	- 181 (59.7) 19 (6.3) 103 (34.0)
	**Intravenous analgesics preferred in the early postoperative period (in the first 2 hours)** Fentanyl Morphine Tramadol Paracetamol Ketamine Other	- 49/253 (16.2/83.8) 51/251 (16.9/83.1) 143/159 (47.4/52.6) 285/17 (94.4/5.6) 19/283 (6.3/93.7) 29/273 (9.6/90.4)
	**The most commonly used regional analgesia methods** Caudal block Lumbar/thoracic epidural Truncal blocks (thorax plane and abdominal wall blocks) Upper extremity blocks Lower extremity blocks	157/82 (65.7/34.3) 88/151 (36.8/63.2) 114/125 (47.7/52.3) 107/132 (44.8/55.2) 59/180 (24.7/75.3)
	**Pain scores** CHEOPS FLACC VAS Wong Baker Face Scale	- 27/249 (9.8/90.2) 66/210 (23.9/76.1) 179/97 (64.9/35.1) 125/151 (45.3/54.7)
	**Medical staff assessing pain** Doctor Nurse Anesthesia student	- 89/193 (31.6/68.4) 234/45 (83.9/16.1) 2/280 (0.7/99.3)

CHEOPS, Children’s Hospital of Eastern Ontario Pain Scale; FLACC, face, legs, activity, cry, consolability; VAS, visual analogue scale.
